# A retrospective validation study of sentinel lymph node mapping for high-risk endometrial cancer

**DOI:** 10.1007/s00404-019-05085-0

**Published:** 2019-02-12

**Authors:** Tian Wang, Yuanjing Hu, Ya He, Peisong Sun, Zhengchen Guo

**Affiliations:** 1 Tianjin Central Obstetrics and Gynecology Hospital, No 156, Nankaisan Road, Nankai District, Tianjin, China; 2 Medical University of Tianjin, No. 22, Qixiangtai Road, Heping District, Tianjin, China

**Keywords:** Endometrial carcinoma, Sentinel lymph node, Indocyanine green, High risks, Lymph-node metastasis

## Abstract

**Objectives:**

To determine the feasibility and performance of sentinel lymph-node (SLN) mapping among women with high-risk endometrial cancer (EC).

**Materials and methods:**

Ninety-eight patients at high-risk EC were enrolled in this retrospective surgical trial from August 2016 to August 2018. All patients underwent intraoperative SLN biopsy, with ICG injection for laparoscopic staging; this was followed by pelvic and paraaortic lymphadenectomy (LAD). Outcomes included SLN detection rate, false-negative SLN algorithm rate, and the negative predictive value (NPV) of the SLN algorithm. The Chi-square test was used to analyze the relationship between SLN mapping and the risk factors. Then, we performed Kappa consistency check (*P* < 0.05 with Meaning), to estimate the consistency of SLN and lymph-node metastasis.

**Results:**

Successful biopsy occurred in 94 patients (170 sides) among 98 patients (196 sides). At least 1 SLN was identified in 86.7% (170/196). Overall, the false-negative rate (FNR) was 11.8% (2/17), NPV was 97.3% (72/74), and sensitivity was 88.2% (15/17). 22/98 patients (22.4%) with high-risk EC had at least one metastatic lymph node identified. When the SLN algorithm was retrospectively applied, the FNR was 9.1% (2/22) and sensitivity was 90.9% (20/22). Considering the surgeon’s experience, 68 cases of EC (except for 30 patients), the detection rate was 89.7% (122/136), NPV was 98.1% (50/51), and the FNR was 5.6% (1/18). The factor significantly affecting the detection rate of SLNs was lymphovascular space invasion (LVSI) (*P* = 0.016). SLN metastasis of EC was associated with depth of myometrial invasion (*P* = 0.034). The analysis result of SLN and the consistency of pelvic lymph-node metastasis status. As detected by Kappa coefficient was 0.939 (*P* < 0.001), suggests highly consistency.

**Conclusions:**

Our SLN detection rate for high-risk EC was the same as previously reported. When SLN is not detected, better after 30 patients’ experience, is a reasonable alternative to complete LAD in high-risk EC. In addition, SLN shows high co-occurrence with pelvic lymph nodes. Therefore, SLN biopsy can be used to diagnose high-risk EC.

## Introduction

Endometrial cancer (EC) is one of the most prevalent among gynecological malignant tumors, and has shown a steady increase in incidence; whether LAD is suitable for EC is a topic of ongoing debate. Standard treatment recommended by relevant guidelines includes a complete or selective pelvic and paraaortic LAD, with accurate identification of nodal status that would help to tailor the postoperative adjuvant treatment. However, the recent studies demonstrated no therapeutic benefit of complete LAD in patients with the early stage EC [1]. As we know, lymph-node resection can cause postoperative complications such as lymphocyst, pelvic infection, and nerve injury [2]. Furthermore, it has been reported that, in the early stages of EC, low-risk factors (1.4–3%) for pelvic lymph-node metastasis were less prominent than high-risk factors (6.4–23%) [3, 4]. In addition, the lymphatic system is a complex structural network, so the removal of all the draining lymph nodes cannot be guaranteed. Moreover, lymph nodes are immune organs, and the removal of regional lymph nodes weakens the anti-tumor immunity of the body. Currently, the lymph nodes that have not yet been transferred have the defensive function of blocking the proliferation of cancer cells, and in the future, immunotherapy is bound to be an important means of tumor treatment [5]. Therefore, in the context of precise and individualized immunotherapy, a technique is needed to accurately assess the state of the lymph nodes without lymph-node resection.

Sentinel lymph node (SLN) is the first node in the primary tumor drainage area and is the most easily metastasized one among the regional lymph nodes. There is increasing evidence supporting the role of SLN biopsy in EC. Published studies have described SLN detection rate to be as high as 85–100% with NPV being 90–99% [6, 7]. Unsurprisingly, this technique has been first recommended as the priority treatment in the 2014 National Comprehensive Cancer Network (NCCN) staging guidelines and EMSO–ESGO–ESTRO Consensus conference on EC [8]. Most studies, however, include patients at low risk for lymph-node involvement and may underestimate FNR. To consider SLN biopsy alone in women with high-risk EC, some validation studies requiring comprehensive surgical staging were needed.

We herein aimed to evaluate the detection rate, sensitivity, NPV, FNR, and SLN positions using ICG- and CSF-guided diagnosis in high-risk EC patients undergoing laparoscopic staging. Then, we also estimated the influence of SLN mapping by univariate analysis to evaluate its feasibility in women at high risk of EC.

## Materials and methods

### Patients

We performed a retrospective analysis of all the patients with EC undergoing ICG SLN mapping at the Tianjin Central Obstetrics and Gynecology Hospital between August 2016 and August 2018. The study was approved by the institutional review board, and all patients signed informed consent. Among 132 patients, 98 patients were eligible who had high-risk endometrial cancer including serous, clear cell, FIGO grade 3 endometrioid, or carcinosarcoma histology based on postoperative pathology. Patients with biopsy-proven cervical involvement or FIGO grade 1/2 endometrioid tumors with deep myometrial invasion were also eligible. Routine preoperative work-up included a transvaginal sonogram, magnetic resonance imaging (MRI), tumor markers, and analysis of the pathological nature of the lesion. At the beginning of the operation, ICG was injected at 3 o'clock and 9 o'clock positions of the cervix [injected slowly (1 cm) to a shallow depth (0.1–0.3 cm)]. SLNs were the earliest to show a high level of fluorescence signal, which was identified using the Pinpoint near-infrared imaging system (Novadaq, Model: PC9000). The red and blue patterns could be seen in the green nodules, and the red core group could be seen to confirm SLNs again. Surgeons remove SLNs and suspected enlarged lymph nodes, and then sent to routine pathological examination. Thereafter, all patients underwent surgical staging, including hysterectomy, bilateral salpingo-oophorectomy and pelvic with or without paraaortic PAD or omentum resection. After the operation, the paraffin-embedded samples were sent for pathological examination (hematoxylin–eosin staining, immunohisto-chemistry). The location of the lymph node, number of nodes, SLN mapping time, and conventional pathological results of SLN were recorded in all patients. The location and number of all retroperitoneal lymph nodes and their pathological results were recorded, as well.

The performance characteristics of SLN mapping alone were retrospectively evaluated and compared with characteristics yielded by applying the algorithm for SLN mapping of EC developed at the Memorial Sloan-Kettering Cancer Center (MSKCC). The SLN algorithm requires (1) peritoneal and serosal evaluation and washing, (2) retroperitoneal evaluation, including the excision of all mapped SLNs and removal of all suspicious nodes regardless of mapping, and (3) if there is no mapping within the hemipelvis, a side-specific pelvic, common iliac, and interiliac LAD should be performed.

### Statistical analysis

Clinicopathologic characteristics were evaluated using the basic descriptive statistics. Overall detection rates, sensitivity, and the NPV of SLN mapping were calculated. A univariate analysis of the risk factor for SLN performance was conducted using SPSS 22.0 statistical software. The counting data were adopted, and then, Kappa consistency check was performed, with *P* < 0.05 implying statistical significance.

## Results

Between August 2016 and August 2018, SLN mapping was performed for 132 patients; of these, 34 patients were excluded, because the patient consented only for SLN removal. The remaining 98 were included in the final analysis. The demographic characteristics of the included patients are shown in Table 1. The mean age of all participants was 53 years (range 37–69 years) with a mean body mass index of 25.4 kg/m^2^ (range 21.2–33.5 kg/m^2^). Data on SLN mapping are summarized in Table 2. The median time from ICG injection into the cervix and intraoperative SLN detection was 7 min (range 0–16 min), On SLN mapping, successful biopsy occurred in 94 patients (170 sides) among 98 patients (196 sides). At least 1 SLN was identified in 86.7% (170/196). 76/98 (77.6%) had it bilaterally in the pelvic region. In the above 98 cases, A total of 283 SLNs were mapped in 94 cases. Malignancy was identified in 20/283 (7.1%) of all SLNs and 16/3491 (0.5%) of all non-SLNs (*P* < 0.001). 22 of the 98 (22.4%) successfully mapped cases showed metastasis to SLN, pelvic LN, and aortic nodes.

**Table 1:** Demographic characteristics of patients

Parameter	Median	Range
Age (years)	53	37–69
BMI (kg/m^2^)	25.4	21.2–33.5
Parameter	*N*	%
Age		
< 60	75	76.8
≥ 60	23	23.2
FIGO stage		
IA	37	37.6
IB	39	40.0
IIIC1	20	20.4
IIIC2	2	2.0
Histology		
Endometrioid	72	73.5
Serous	10	10.2
Clear cell	12	12.2
Undifferentiated	4	4.1
Grade		
I–II	43	59.7
III	29	40.3
Lymphovascular space invasion		
Present	42	42.9
Absent	56	57.1
Myometrial invasion		
≥ 50%	43	43.9
< 50%	55	56.1
Tumor size		
≥ 2 cm	77	78.6
< 2 cm	21	21.4

**Table 2:** SLN mapping characteristics

Time from ICG injection to SLN identification, min, mean (range)	7 (0–16)
Number of right SLN per patient, mean (range)	1.8 (0–6)
Number of left SLN per patient, mean (range)	1.2 (0–3)
SLN detection rate, *n* (%)	
Overall	94 (86.7)
Bilateral	76 (77.6)
Failed	4 (4.1)
SLN location, *n* (detected numbers of side/total numbers of side %)	
External iliac	101 (101/283.36%)
Internal iliac	45 (45/283.16%)
Obturator	99 (99/283.35%)
Common iliac	29 (29/283.10%)
Periaortic	9 (9/283.3%)
Patients with lymph-node metastases	22 (22.4%)
Median number of PLND (range)	23 (11–40)
Median number of PALND (range)	14 (5–26)

*ICG* indocyanine green, *SLN* sentinel lymph node, *PLND* pelvic lymph-node dissection, *PALND* paraaortic lymph-node dissection

There were five patients who were non-SLN-positive for the pelvis, and no SLN was detected on the same side. 17 patients had ≥ 1 SLNs removed because of metastasis; 7/17 patients (41.2%) with metastatic SLNs also had positive non-SLNs. Metastases were limited to SLNs in 10 cases (58.8%), and were observed only in non-SLNs in 2 cases, which was labeled as a true–false, thus accounting for a false-negative rate of 2/17 (11.8%), the NPV was 97.3% (72/74), and the sensitivity was 88.2% (15/17). When the SLN algorithm was retrospectively applied, the FNR was 9.1% (2/22) and the sensitivity was 90.9% (20/22). The distribution of SLNs is as follows: external iliac, 101 (101/283.36%); internal iliac, 45 (16%); obturator, 99 (35%); common iliac, 29 (10%); and periaortic, 9 (3%).

The study showed that surgeons had at least surgical experience of 30 cases. Among the 98 cases, the detection rate was 89.7% (122/136), NPV was 98.1% (50/51), and the FNR was 5.6% (1/18) in 68 patients. In addition, the univariate analysis of the influential factors of SLN mapping was conducted (Table 3). It showed that the lymphovascular space invasion was associated with SLN mapping (*P* = 0.016). In addition, no obvious correlation factors, such as pathological type, grade, depth of myometrial invasion, and lymphovascular space invasion, were identified (*P* > 0.05). There was a significant correlation between lymph-node metastasis of EC and depth of myometrial invasion (*P* = 0.034) (Table 4). The analysis of the consistency of SLNs and pelvic lymph-node metastasis status identified the Kappa coefficient to be 0.939 (*P* < 0.001), suggesting high consistency (Table 5).

**Table 3:** Univariate analysis of the influence factors of SLN mapping

Variable	*n* (%)	Mapping [*n* (%)]	*P*
Age			
< 60	75 (76.8)	130 (86.7)	0.960
≥ 60	23 (23.2)	40 (88.5)	
Histology			
Endometrioid	72 (73.5)	122 (84.7)	0.253
Other types	26 (26.5)	48 (91.7)	
Grade			
I–II	43 (59.7)	77 (89.5)	0.051
III	29 (40.3)	45 (77.8)	
Lymphovascular space invasion			
Present	42 (42.9)	79 (94.0)	0.016
Absent	56 (57.1)	91 (81.3)	
Myometrial invasion			
≥ 50%	43 (43.9)	73 (84.9)	0.499
< 50%	55 (56.1)	97 (88.5)	
Tumor size			
< 2 cm	21 (21.4)	38 (90.5%)	0.582
≥ 2 cm	77 (78.6)	132 (85.7)

**Table 4:** Univariate analysis of the influence factors of lymph-node metastasis

Variable	*n* (%)	Metastasis [*n* (%)]	*P*
Age			
< 60	75 (76.8)	15 (20.0)	0.294
≥ 60	23 (23.2)	7 (30.4)	
Histology			
Endometrioid	72 (73.5)	14 (19.4)	0.236
Other types	26 (26.5)	8 (30.8)	
Grade			
I–II	43 (59.7)	10 (23.3)	0.102
III	29 (40.3)	12 (41.4)	
Lymphovascular space invasion			
Present	42 (42.9)	10 (94.0)	0.780
Absent	56 (57.1)	12 (41.4)	
Myometrial invasion			
≥ 50%	43 (43.9)	14 (84.9)	0.034
< 50%	55 (56.1)	8 (88.5)	
Tumor size			
< 2 cm	21 (21.4)	7 (90.5%)	0.177
≥ 2 cm	77 (78.6)	15 (85.7)

**Table 5:** Analysis of the consistency of SLN and pelvic lymph-node metastasis

SLN	Pelvic lymph node		Total
	*P*	*N*	
*P*	20	0	20
*N*	2	72	74
Total	22	72	94

Kappa coefficient 0.939, *P* < 0.001

## Discussion

SLN biopsy was first introduced in 1977 with a study of penile cancer in Cabana, Paraguay. Currently, changed diagnosis as metastasis to SLN would help to diagnose (and not treat) breast cancer. A thorough clinical study was conducted with regard to skin malignant melanoma and vulvar cancer, and satisfactory clinical results were obtained [9]. Since lymphatic mapping including identification of SLNs in EC was first described in 1996, surgical staging has been controversial [10]. Systemic therapy is indicated for women with extra-pelvic disease, with therapeutic decisions directly determined based on the identification of nodal and distant metastases. If lymph-node status is unknown, there is a possibility that some patients are overtreated or undertreated. Given the low risk of lymph-node metastases in the early stage disease, performing a pelvic and paraaortic LAD in every patient may be more harmful than helpful. Currently, SLN analysis is a new and innovative method to treat the early stages of EC [11].

In gynecological cancers, SLN mapping with 99Tc, blue dye, and ICG in conjunction with a near-infrared (NIR) fluorescence imaging system has been reported. In a meta-analysis, Bodurtha Smith et al. analyzed 4915 patients from 51 studies, and the total detection rate of SLN achieved mostly using blue dye and 99Tc was 81% and the sensitivity was 96% [2]. Buda et al. reported an overall and bilateral detection rate of 89% and 54%, respectively, using blue dye [6]. Lin et al. [12] reported a meta-analysis which included 44 studies encompassing 2236 cases. After statistical analysis, the overall detection rates and sensitivity of ICG were 93% and 91%, respectively, with an NPV of 96.4%. Therefore, SLN mapping with ICG cervical injection has a high success rate in SLN detection (90–100%) [13–16].

Considering that the uterus is located deep in the pelvic cavity and bilateral lymphatic drainage were performed on the side. As the SLN detection rate and accuracy are high enough, they can comprehensively evaluate the situation of regional lymph node involvement and be used as an alternative standard treatment. In this retrospective validation study, at least one metastatic lymph node was identified in 22/98 patients (22.4%). SLN mapping was successful in 94 patients (86.7%). We report an FNR of 11.8% and NPV of 97.3%. More recently, Ehrisman et al. [16] developed an algorithm that includes performing a side-specific pelvic LAD when an SLN is not detected. Retrospective implementation of this algorithm resulted in a significant decrease in their FNR from 7.7% to 0% in women at high risk of EC. When the SLN algorithm was retrospectively applied in our study with 30 patients’ experience, the FNR was 5.6% (1/18) and NPV was 98.1% (50/51). Our NPV is comparable to rates previously reported for the early stages of EC [13–16]. Our outcome suggests that the implementation of an EC surgical algorithm along with SLN mapping and biopsy is a successful method of identifying the metastatic nodal disease in the majority of patients at high risk for EC.

**Fig. 1:**
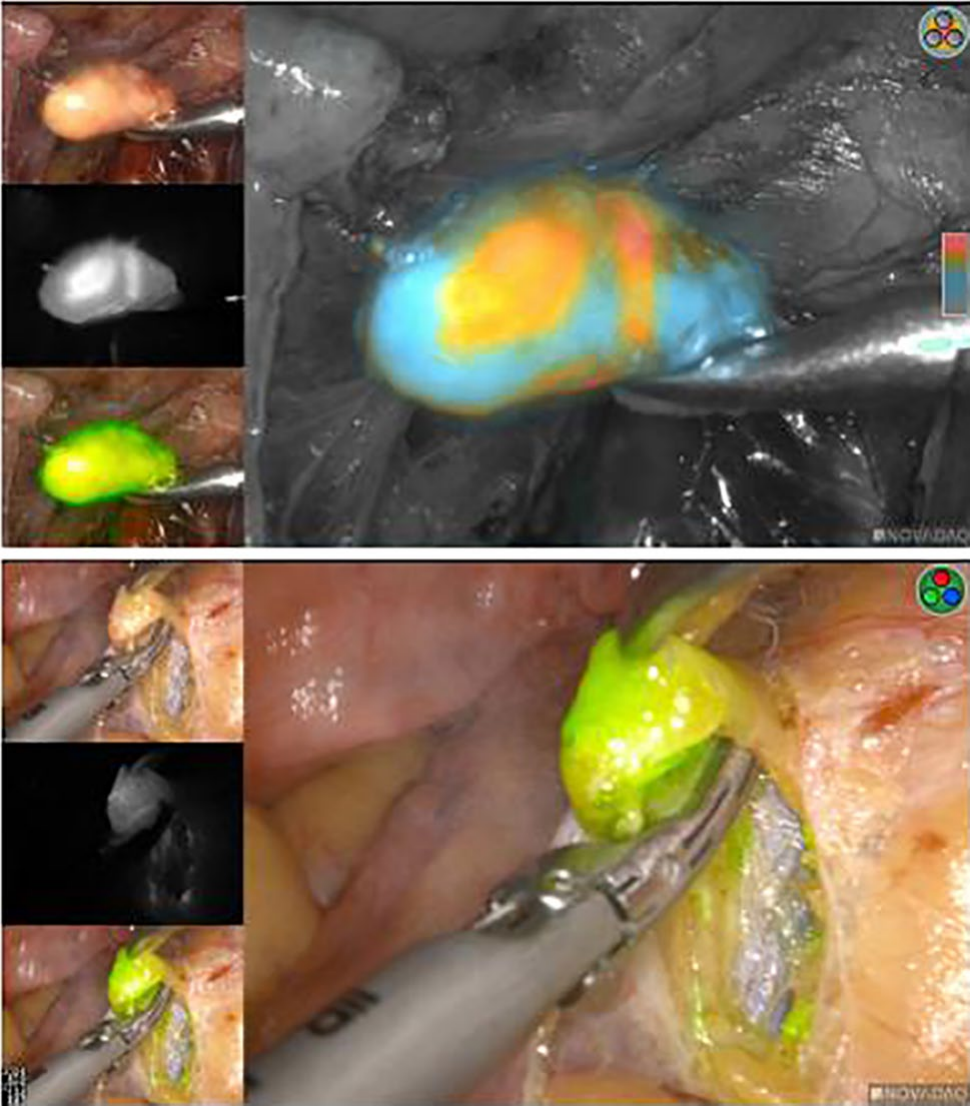
Imaging of SLNs in different modes

We believe that the good results recorded in our series are largely attributed to the tracer and CSF used. During the operation, we switched three types of images (Fig. 1) to identify the presence or absence of SLN, such as black and white, fluorescence, and CSF, to prevent adipose tissue or lymphatics from being mistaken for SLN. CSF may help surgeons to identify true SLN from fatty tissues which absorb fluorescent dye does, but do not contain the true nodal tissue. The key ranges from low levels of ICG uptake (gray) to the highest rate of ICG uptake (red).

As mentioned before, CSF can help us to correctly find SLNs; however, if we know its drainage standard, the accuracy of SLN detection will substantially increase. In the recent studies [17, 18], it has been shown that SLN drainage pathways have certain regularities. The pelvic anterior SLN drainage has three access points: (1) the upper cervical bypass (UPP), which is along the uterine artery, leading the medial and (or) obturator lymph nodes of the external iliac; (2) next to the cervix (LPP), and (3) the pelvic funnel ligament pathway IPP. Lymph-node metastasis is most likely to occur via the UPP pathway. In our research, the external iliac and obturator lymph nodes were 200, accounting for 71% of the SLNs detected. Most SLNs were found located in the UPP circulation, which is consistent with the literature.

The relevant literature has reported that low- and high-risk factors for pelvic lymph-node metastasis were 1.4–3% and 6.4–23%, respectively [2, 11]. The SLN detection in the abdominal aorta was less than 5% [19]. In addition, the probability that SLN had an abdominal aortic lymph-node transfer was low at 0.8–1%, with a 3% chance of metastatic EC in high-risk patients [20]. In our study, 22.4% (22/98) of patients had pelvic lymph-node metastases. 3% (9/283) of SLNs were detected and 2% (2/98) of patients underwent paraaortic lymph nodes. In addition, SLN metastasis of EC was associated with depth of myometrial invasion (*P* = 0.034). As reported in the literature [2, 16], patients with high-risk factors can show reduced lymph-node detection rate and increased lymph-node metastasis. The other factors related to the success of SLN mapping are as follows: the detection method, tracer used, patient factors (obesity and dissection), and experience of surgeons [21]. Based on our study, the major factor significantly affecting the detection rate of SLN was LVSI (*P* = 0.016). However, the experience of surgeons is very important, as well; studies show that a surgeon needs to have performed the procedure at least 30 times for proficiency [22]. In our study, 68 cases of EC (except for 30 patients), the detection rate was 89.7% (122/136), NPV was 98.1% (50/51), and the FNR was 5.6% (1/18). Therefore, the factors affecting SLN mapping include surgeon’s experience. We need to avoid all the factors as much as possible, only in this way can sentinel lymph nodes be more valuable in clinical application.

Concerning the two confirmed true–false case, negative pelvic SLN was identified, and the positive malignant non-SLN was removed from the contralateral common iliac or the abdominal aortic lymph nodes. The two patients had three risk factors, including tumor size > 4 cm, deep depth of myometrial invasion, and lymphovascular space invasion. The reasons for the presence of false-negatives may be as follows: (1) the deep depth of myometrial invasion indicated that the tumor growth time was longer, the tumor cells had increased metastasis, and the tumor blocked the lymphatic channel, thus altering the original lymph circulation, and SLN was concentrated in the lymph nodes without metastasis. (2) False-negatives can also be caused by inaccurate SLN position. Persson [18] reported that pelvic SLNs were divided into three categories: in the presence of an ICG-positive afferent lymph vessel, the ICG-negative node draining this lymph vessel was defined as SLN type 2. The true–false patient showed that, after ICG injection, SLN developed in multiple parts. In theory, ICG was absorbed by lymphoid epithelial cells to simulate lymphatic metastasis and there can be multiple lymphatic pathways that develop at the same time, even though there is only one true SLN in one pathway. If it is necessary to detect the presence of a solid lymph node to avoid the ‘SLN type 2’ being left out in any developing lymphatic pathway. SLNs are detected as false-negatives, and also have kinds of subjective reasons, as long as at the beginning of the strict indication, in strict accordance with the standard operating procedures, intraoperative careful earnest, strengthen the SLN pathology examination detected, even in some high FNR at the beginning, but after a period of practice, can reduce the FNR to an acceptable level. Herein, long-term studies will be needed to determine the impact of SLN biopsy alone on survival of women with high-risk EC.
